# A focus on the future of opioid prescribing: implementation of a virtual opioid and pain management module for medical students

**DOI:** 10.1186/s12909-021-03058-z

**Published:** 2022-01-06

**Authors:** Jenna R. Adalbert, Asif M. Ilyas

**Affiliations:** 1 Sidney Kimmel Medical College at Thomas Jefferson University, Philadelphia, USA; 2 Jefferson College of Population Health, Thomas Jefferson University, Philadelphia, PA USA; 3 Rothman Orthopaedic Institute Foundation for Opioid Research & Education, Philadelphia, USA

**Keywords:** Medical education, Virtual curriculum, Opioid prescribing, Pain management, Opioid epidemic

## Abstract

**Background:**

The United States opioid epidemic is a devastating public health crisis fueled in part by physician prescribing. While the next generation of prescribers is crucial to the trajectory of the epidemic, medical school curricula designated to prepare students for opioid prescribing (OP) and pain management is often underdeveloped. In response to this deficit, we aimed to investigate the impact of an online opioid and pain management (OPM) educational intervention on fourth-year medical student knowledge, attitudes, and perceived competence.

**Methods:**

Graduating students completing their final year of medical education at Sidney Kimmel Medical College of Thomas Jefferson University were sent an e-mail invitation to complete a virtual OPM module. The module consisted of eight interactive patient cases that introduced topics through a case-based learning system, challenging students to make decisions and answer knowledge questions about the patient care process. An identical pre- and posttest were built into the module to measure general and case-specific learning objectives, with responses subsequently analyzed using the Wilcoxon matched-pairs signed-rank test.

**Results:**

Forty-three students (19% response rate) completed the module. All median posttest responses ranked significantly higher than paired median pretest responses (*p* <  0.05). Comparing the paired overall student baseline score to module completion, median posttest ranks (Mdn = 206, IQR = 25) were significantly higher than median pretest ranks (Mdn = 150, IQR = 24) (*p* <  0.001). Regarding paired median Perceived Competence Scale metrics specifically, perceived student confidence, capability, and ability in opioid management increased from “disagree” (2) to “agree” (4) (*p* <  0.001), and student ability to meet the challenge of opioid management increased from “neither agree nor disagree” (3) to “agree” (4) (*p* <  0.001). Additionally, while 77% of students reported receiving OP training in medical school, 21% reported no history of prior training.

**Conclusion:**

Implementation of a virtual, interactive module with clinical context is an effective framework for improving the OPM knowledge, attitudes, and perceived competence of fourth-year medical students. This type of intervention may be an important method for standardizing and augmenting the education of future prescribers across multiple institutions.

**Supplementary Information:**

The online version contains supplementary material available at 10.1186/s12909-021-03058-z.

## Background

The United States (U.S.) opioid epidemic is an ongoing public health crisis, fueled in part by inaccurate prescriber beliefs of low opioid harm and addiction risks [1]. Yet following recognition of opioid risks, 80% of the world’s entire opioid supply is still consumed annually in the U.S., which constitutes merely 5% of the global population [2]. In 2018, 46,802 overdose deaths (69.5% of all drug overdose deaths) in the U.S. were attributed to opioids, with prescription opioid overdoses comprising almost 32% of these deaths [3]. Accordingly, correcting prescribing practices seems to be an important potential solution for harm reduction, but provider uncertainty in accurately assessing patient pain and prescribing appropriate opioid amounts remains a prominent barrier [4].

As members of the next generation of prescribers, medical students are important agents of impact on the trajectory of the U.S. opioid epidemic. Part of the educational approach to reduce inappropriate opioid prescribing (OP) has included the incorporation of pain assessment, pain management (PM), and substance use disorder treatment into medical school curricula in response to calls from regulating bodies such as the Association of American Medical Colleges [4, 5]. However, the response of medical schools in adapting curricula to train medical students has been underwhelming in the context of the crisis severity, limited by a lack of standardized curricula and adequately trained faculty to teach and assess learning surrounding these concepts [4, 6]. Given this identified deficit in preparing medical students for their impending role as prescribers, creating a resource to address gaps in opioid and pain management (OPM) knowledge is an educational imperative.

The purpose of this study was to investigate the impact of an online educational intervention targeting fourth-year medical student knowledge, attitudes, and perceived competence in OP and PM. The study was designed as an interactive module purposed to reinforce student understanding of the opioid epidemic and prescriber contribution, explore the risks, benefits, and role of opioids in combination with alternative analgesics for PM, introduce evidence-based prescribing guidelines and tools for opioid stewardship such as the Prescription Drug Monitoring Program (PDMP) or morphine milliequivalent (MME) conversion resources, and provide practice with OP and PM decision-making in a variety of clinical scenarios. The goal was to assess the efficacy and success of this virtual intervention in delivering key learning objectives on opioids and PM while improving the delivery of these topics in our own curricula.

## Methods

### Participants and study procedures

Prior to module dissemination, we recruited students from the dual-degree Doctor of Medicine and Master of Public Health (MD/MPH) training program (*n* = 10) in between their third and final year of medical school at the Jefferson College of Population Health to pilot the learning activity. After incorporating feedback, we sent graduating students completing their final year of medical education at Sidney Kimmel Medical College of Thomas Jefferson University and intending to begin training at a residency program in 2021 (*n* = 228) an e-mail invitation to complete an online OPM module. Completion of this learning activity was completely voluntary with a de-identified pre- and posttest built into the module to assess its effectiveness at delivering educational content. We sent reminders to complete the module twice by email over a five-week period.

### Module design and content

We developed content for the module using human-centered design [7] to create patient-oriented scenarios that achieved specific learning objectives and agile project management for the process of iterative drafts and feedback. J.A. designed each case draft in Microsoft Word and sent several revisions to A.I. until both authors agreed that the cases successfully achieved their individual learning objectives. A.I. then designed the case vignettes for each module to challenge students to practice higher-level provider prescribing decisions and sent them to J.A. for feedback on clarity from the student perspective. J.A. translated the cases and their respective vignettes into an online platform at the end of this exchange. Upon completion of this process, the module was disseminated to our student pilot group (*n* = 10) with open-ended feedback requested for each case, as well as final overall feedback at the end of the module to improve content and clarity for future student learners. After receiving feedback, edits were incorporated by J.A. and reviewed by A.I. before dissemination to the fourth-year medical student class (*n* = 228).

Our final module consisted of eight interactive, hypothetical patient cases and encompassed a wide range of fundamental OPM topics. Instead of frontloading students with lecture material, we introduced educational topics through an interactive case-based learning system [8], challenging learners to make decisions and answer knowledge questions about the patient care management process. We designed patient cases sequentially with key concepts repeated throughout the module to promote reinforcement of learning objectives and practice applying new concepts to patient management. Examples of our interactive design included “true or false” questions on topics such as the opioid epidemic or drug mechanisms of action, multiple choice questions exploring the “next best step” in patient care, and choosing verbal responses to patient questions or concerns. We followed each interactive experience immediately with feedback on answer choice selection to reinforce student learning. Patient cases that we created for the module to address key OPM topics included:

1. An 85-year-old woman with advanced dementia and joint pain from osteoarthritis to learn about pain assessment and management complexities in older adults.
2. A 61-year-old man with low back pain and depression to learn about strategic approaches to the treatment of chronic pain, proper opioid disposal practices to prevent diversion, and the role of mood disorders in chronic pain outcomes.
3. A 49-year-old man with shoulder pain on chronic opioid therapy to learn about MME conversions, key principles for initiating and modifying opioid treatment, and the role of co-prescribing naloxone.
4. A 31-year-old woman with trauma injuries and a history of opioid use disorder (OUD) to learn about medication-assisted treatment and the challenges of acute PM in patients with opioid tolerance.
5. A 42-year-old man with a radial fracture to learn about postoperative opioid management, common types of outpatient opioid prescriptions, and the role of the PDMP.
6. A 10-year-old boy with a humerus fracture to learn about pediatric PM and medication dosing, and the risks of OP in the adolescent population.
7. A 29-year-old pregnant woman with sickle cell disease on chronic opioid therapy to learn about safe PM in pregnancy and postnatal effects of opioids.
8. A 37-year-old woman with breast cancer to learn about pain syndromes in cancer survivors and the role of opioids in chronic cancer pain.

**Fig. 1:**
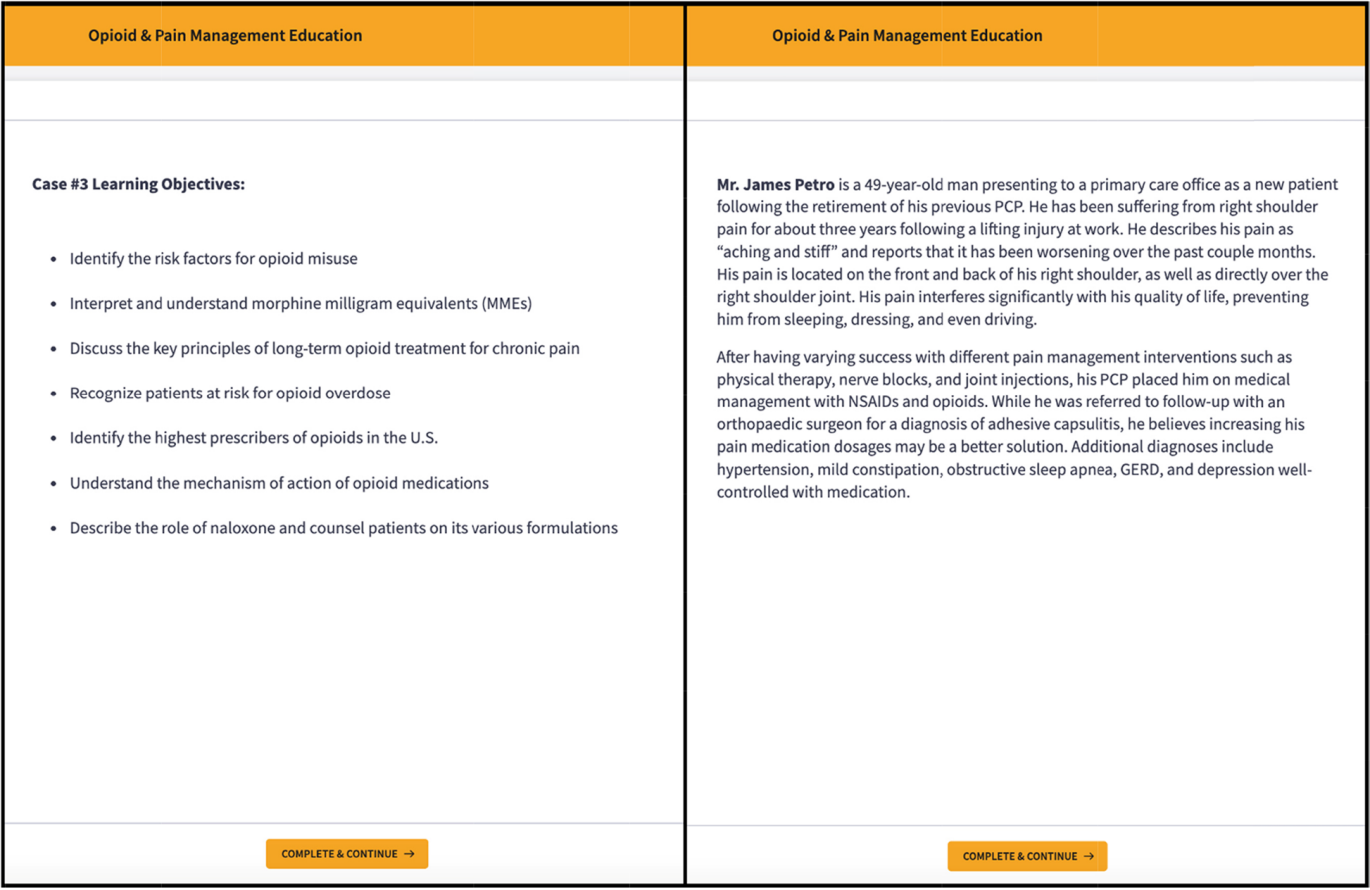
Examples of slides from Case #3 including the learning objectives and case introduction

**Fig. 2:**
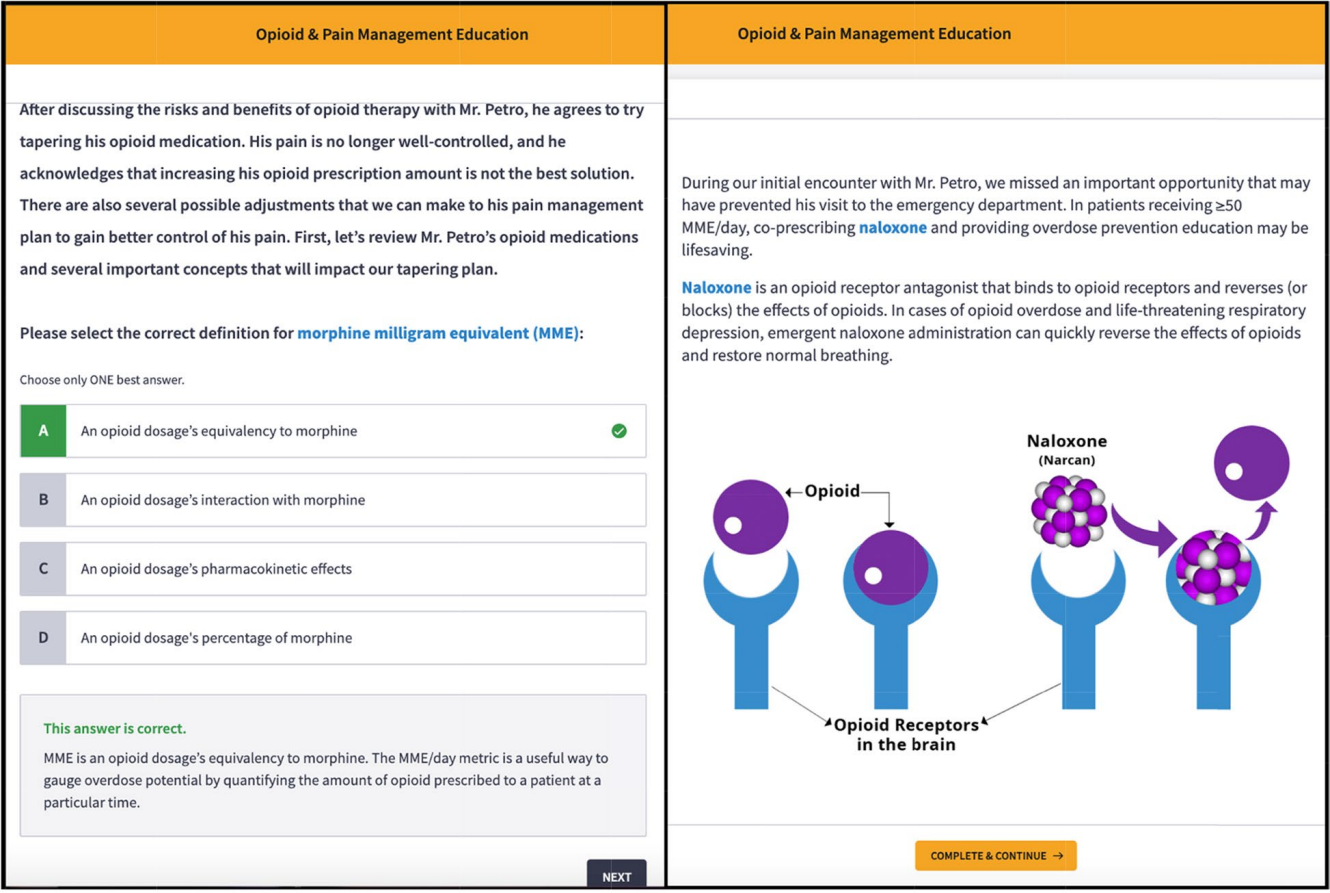
Examples of slides from Case #3 including an interactive multiple-choice question and case progression with illustration

Figures 1 and 2 provide a “snapshot” of several slides from Case #3 to demonstrate the cartoon and text modalities used to design each module.

**Fig. 3:**
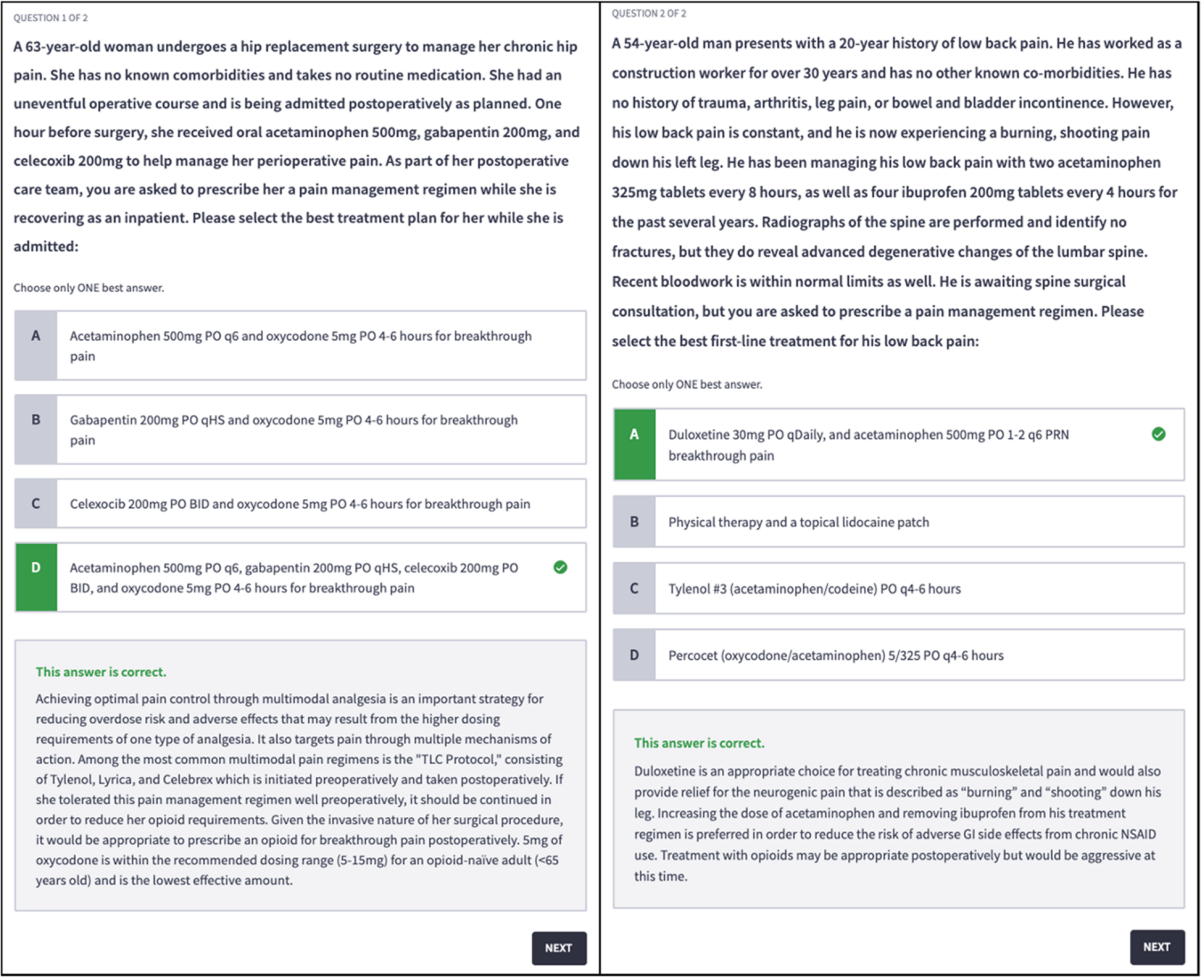
Examples of two “Prescriber Practice” case vignettes included at the end of each patient case

Additionally, we provided two additional case vignettes entitled “Prescriber Practice” at the end of each patient case to follow and reinforce the concepts (*n* = 16), challenging students to perform intern-level prescribing decisions (Fig. 3).

On average, learners spent a total of three hours completing the module, with each case lasting approximately 20-30 min. All enrolled students completed the course, and no incentive was provided to encourage course completion. In the initial feedback session, our pilot group reported that the module was completed in two to three sittings, and pre/posttest time stamps for each student in both the pilot and final student samples supported these reported intervals. We built the module on an online course platform that enabled students to create an account and work incrementally.

The entire course can be accessed at: https://rothman-opioid-education.thinkific.com/.

### Performance assessment

For accurate reporting of student changes from baseline, we designed the pre- and posttest identically aside from an introductory question on the pretest exploring prior student experience with OP (i.e. medical school, extracurriculars, personal reading, etc.) and a final open-ended question on the posttest providing an opportunity for student feedback to improve module content delivery. Our first question on the pre- and posttest assessed student satisfaction with their level of OP training and was rated on a Likert scale from 1 = “very dissatisfied” to 5 = “very satisfied” (3 = “neutral”). We rated the remaining questions on a Likert scale from 1 = “strongly disagree” to 5 = “strongly agree” (3 = “neither agree nor disagree”). For questions 2-42, students rated their level of agreement with statements designed to evaluate general and case-specific learning objectives (i.e. “I am familiar with safe strategies for disposing of unused opioids”). The final four questions (43-46) were created to assess student perceived competence in OP using a Perceived Competence Scale (PCS) modified to measure domain-specific constructs from the Self-Determination Theory [9]. The PCS is a brief questionnaire based off the Self-Determination Theory, which is a well-validated and reliable instrument for measuring behavior change [10]. The complete pre- and posttest are located in Additional file 1.

### Statistical analysis

We expressed categorical Likert scale variables from the pre- and posttest as numbers (1-5) and compared each question using a Wilcoxon matched-pairs signed-rank test to assess changes in student performance. Given that we structured all questions with rating 5 (“highly satisfied” or “strongly agree”) designated as the most favorable selection, we calculated an overall score on the pre- and posttest for each student and compared these scores using the Wilcoxon matched-pairs signed-rank test to create a general performance distribution. We considered a two-sided *p*-value of less than 0.05 to be statistically significant for our study and performed statistical analyses using SPSS v.25 (IBM, Armonk, NY, USA).

## Results

### Prior sources of opioid training

We recruited forty-three students (19% response rate) to complete the module. We instructed students to select all sources of OP training prior to their completion of the educational module: 33 (77%) reported that training was received in medical school, 16 (37%) reported exposure through personal reading, 13 (30%) reported experience through extracurriculars (i.e. research, volunteering, etc.), 4 (9%) reported exposure through “other” sources, 1 (2%) reported experiences during undergraduate education, and 9 (21%) reported no history of any type of formal training.

### Student knowledge and attitudes

**Fig. 4:**
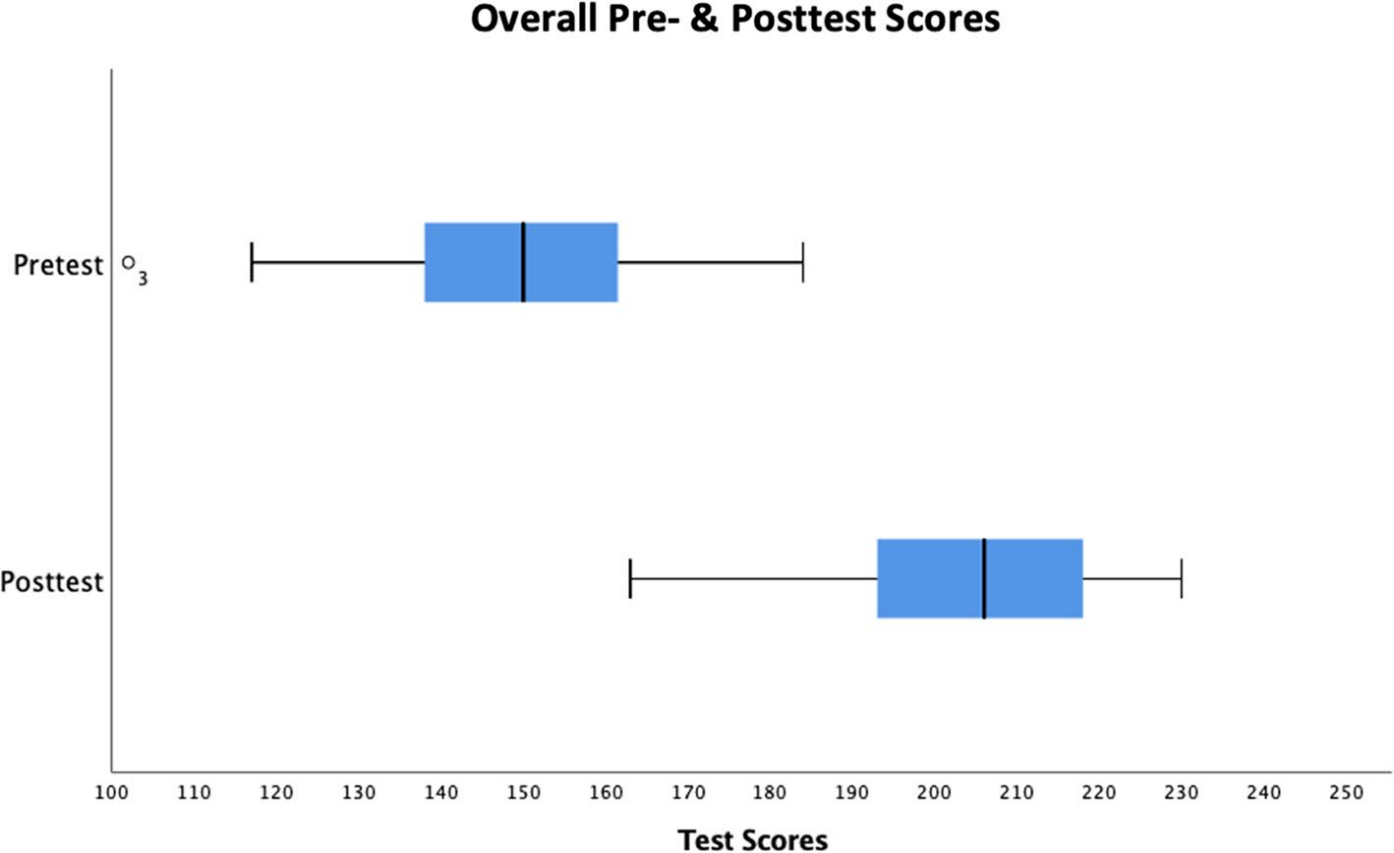
General performance distribution of overall student pre- and posttest scores

Table 1 describes the median of student responses for each question and a median overall score reported as pre- and posttest ranks with an interquartile range to express score variability. All median posttest responses ranked significantly higher than paired median pretest responses at a *p* <  0.05 level of statistical significance, demonstrating improvement in student knowledge and attitudes for each general and case-specific learning objective. Figure 4 illustrates the general distribution of pre- and posttest changes in calculated overall scores. Comparing the paired overall student baseline score to module completion, median posttest ranks (median = 206, interquartile range = 25) were significantly higher than median pretest ranks (median = 150, interquartile range = 24) (*p* <  0.001), indicating individual student improvement from pre- to posttest across a wide range of student baseline knowledge and attitudes. Given the question structuring described (see Statistical Analysis), the pre−/posttest scale ranged from a minimum of 46 to a maximum of 230.

**Table 1:** Pre- and posttest ranks of medical student median scores for each question and overall using the Wilcoxon matched-pairs signed-rank test

Module Pre- & Posttest Ranks			
	Median (Interquartile Range)		
Question	Pretest	Posttest	***p***-value
**Student Background**			
How satisfied are you with the amount of OP training that you have received in your formal education thus far?	3 (2)	4 (1)	< 0.001
**General Learning Objectives**			
I understand the risks of opioids in chronic PM.	4 (0)	4 (1)	< 0.002
I understand the benefits of opioids in chronic PM.	4 (0)	4 (1)	< 0.001
I understand when it is appropriate to prescribe opioids for chronic PM.	3 (1)	4 (1)	< 0.001
I understand the risks of opioids in acute PM.	4 (0)	5 (1)	< 0.001
I understand the benefits of opioids in acute PM.	4 (0)	5 (1)	< 0.001
I understand when it is appropriate to prescribe opioids for acute PM.	3 (2)	4 (1)	< 0.001
I am familiar with the types of opioid medications used for PM.	4 (1)	4 (1)	< 0.001
I am familiar with the types of non-opioid medications used for PM.	4 (0)	5 (1)	< 0.001
I understand the role of prescription opioids in the opioid epidemic.	4 (1)	5 (1)	< 0.001
I am familiar with the Prescription Drug Monitoring Program and know when to use it.	4 (1)	5 (1)	< 0.001
I am familiar with OP guidelines (i.e. dosages and amounts to prescribe).	2 (1)	4 (0)	< 0.001
**Case #1**			
I understand the differences in OP for older adults (> 65) vs. adults (< 65).	2 (1)	4 (1)	< 0.001
I understand the complexities of pain assessment in patients with dementia.	3 (2)	4 (1)	< 0.001
I understand the differences between nociceptive, neuropathic and inflammatory pain.	4 (1)	5 (1)	< 0.001
I am familiar with the risks and benefits of commonly used pain medications for older adults (> 65).	3 (2)	5 (1)	< 0.001
I am familiar with safe PM strategies for older adults (> 65).	3 (2)	4 (1)	< 0.001
**Case #2**			
I understand the differences in treating nociceptive, neuropathic and inflammatory pain.	3 (2)	5 (1)	< 0.001
I am familiar with first-line treatments and strategies for chronic PM.	3 (2)	5 (1)	< 0.001
I understand the concept of “opioid diversion.”	3 (2)	5 (1)	< 0.001
I am familiar with safe strategies for disposing of unused opioids.	2 (2)	5 (1)	< 0.001
**Case #3**			
I am familiar with the risk factors for patient opioid misuse.	4 (1)	5 (1)	< 0.001
I am familiar with safe management strategies for patients on long-term opioid treatment for chronic pain.	2 (1)	4 (1)	< 0.001
I understand the concept of opioid tapering.	4 (1)	4 (1)	< 0.001
I understand the concept of morphine milligram equivalents (MMEs).	4 (2)	5 (1)	< 0.001
I understand how to convert morphine milligram equivalents (MMEs).	2 (3)	5 (1)	< 0.001
I am familiar with the symptoms of an opioid overdose.	4 (1)	5 (1)	< 0.005
I understand the role of naloxone in opioid overdose.	5 (1)	5 (0)	< 0.002
I understand the importance of co-prescribing naloxone with opioids.	4 (1)	5 (0)	< 0.001
**Case #4**			
I understand the role of medication-assisted treatment (MAT) in patients with OUD.	4 (1)	5 (0)	< 0.001
I understand the differences between methadone, buprenorphine, and naltrexone.	4 (1)	5 (1)	< 0.001
I understand the concept of patient-controlled analgesia (PCA).	4 (0)	5 (1)	< 0.001
I am familiar with the differences in opioid dosing requirements for opioid-tolerant vs. opioid-naive patients.	3 (2)	4 (1)	< 0.001
**Case #5**			
I understand the danger of co-prescribing benzodiazepines and opioids.	4 (1)	5 (0)	< 0.001
I understand the role of multimodal analgesia in PM.	4 (0)	5 (1)	< 0.001
**Case #6**			
I understand the differences in pain medication dosing for the pediatric vs. adult population.	2 (1)	4 (1)	< 0.001
I am familiar with the risks of prescribing opioids to adolescents for PM.	4 (2)	4 (1)	< 0.001
**Case #7**			
I am familiar with pain medications that are safe for pregnant patients.	2 (2)	4 (2)	< 0.001
I am familiar with safe opioid management strategies for pregnant patients.	2 (1)	4 (1)	< 0.001
I understand the postnatal effects of opioids on neonates.	4 (0)	5 (1)	< 0.001
**Case #8**			
I am familiar with the concept of pain syndromes in cancer survivors.	3 (2)	4 (1)	< 0.001
I understand the role of opioids in chronic cancer pain.	4 (1)	4 (1)	< 0.001
**Student Perceived Competence**			
I am confident in my ability to manage opioids for patient pain.	2 (1)	4 (0)	< 0.001
I am capable of managing opioids for patient pain.	2 (1)	4 (0)	< 0.001
I am able to provide opioid management for patient pain.	2 (2)	4 (0)	< 0.001
I am able to meet the challenge of opioid management for patient pain.	3 (2)	4 (0)	< 0.001
**Overall score**	150 (24)	206 (25)	< 0.001

### Student perceived competence

**Fig. 5:**
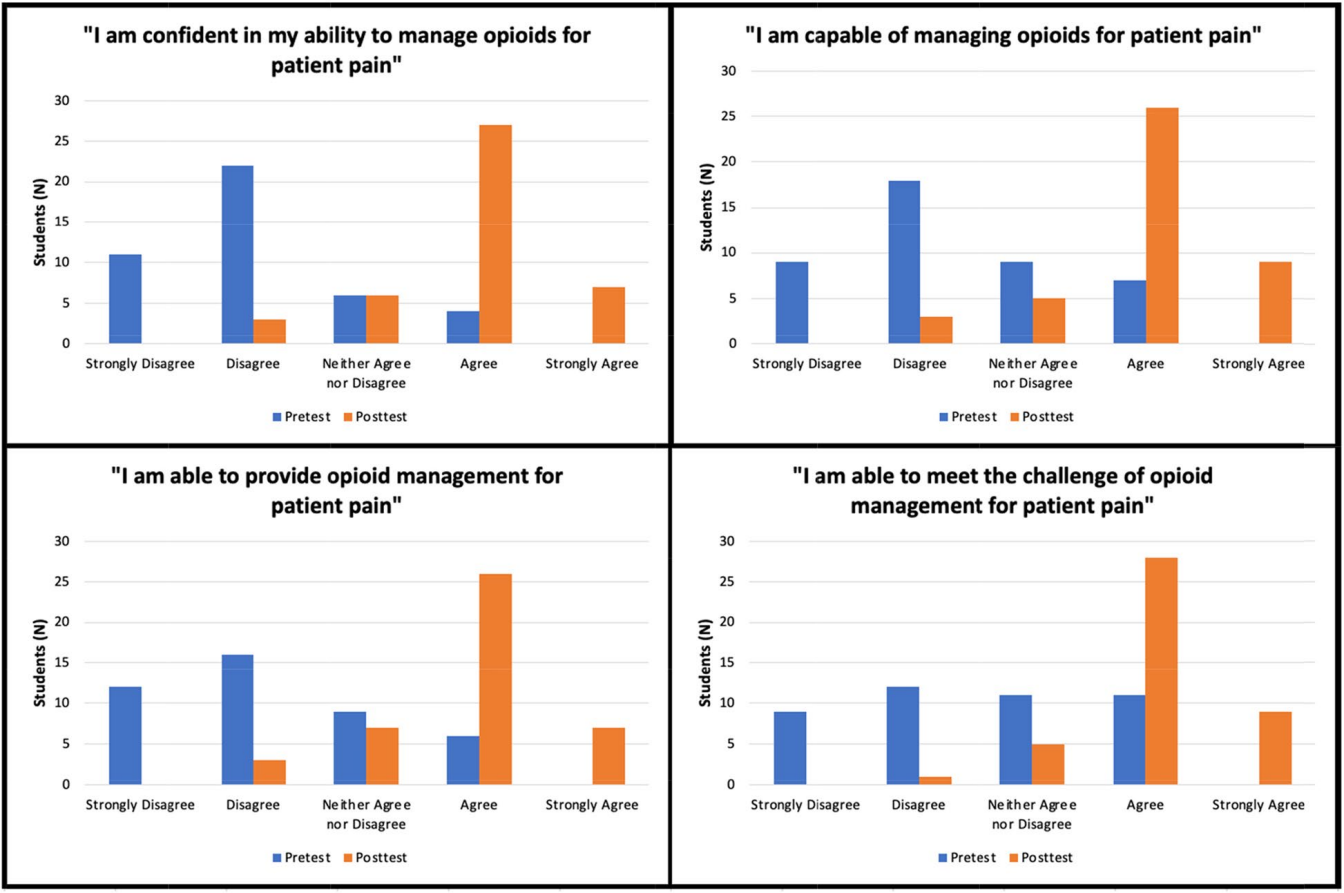
Pre- and posttest changes on a 5-level Likert scale of median medical student perceived competence scores

In Fig. 5, pre- and posttest changes in student perceived competence assessed by the four questions from the modified PCS (“I am confident in my ability…, I am capable…, I am able to provide…, I am able to meet the challenge…”) are described by the number of student responses for each question.

- For perceived student confidence in ability to manage opioids, the majority of students selected “disagree” (*n* = 22, 51%) on the pretest, with a majority change to “agree” (*n* = 27, 63%) on the posttest (Fig. 5). Accordingly, the paired student median response increased from “disagree” (2) to “agree” (4) between the pre- and posttest (*p* <  0.001) (Table 1).
- Regarding perceived student capability of managing opioids, the majority of the students selected “disagree” (*n* = 18, 42%) on the pretest, with a majority change to “agree” (*n* = 26, 60%) on the posttest (Fig. 5). On the pre- and posttest, paired student median responses reflected this increase from “disagree” (2) to “agree” (4) (*p* <  0.001) (Table 1).
- For perceived student ability to provide opioid management, the majority of students selected “strongly disagree” (*n* = 12, 28%) and “disagree” (*n* = 16, 37%) on the pretest, with a majority change to “agree” (n = 26, 61%) on the posttest (Fig. 5). Correspondingly, student paired median responses increased from “disagree” (2) to “agree” (4) (*p* <  0.001) between the pre- and posttest (Table 1).
- Finally, perceived student ability to meet the challenge of opioid management was evenly split on the pretest for selections of “strongly disagree” (*n* = 9, 22%), “disagree” (n = 12, 28%), “neither agree nor disagree” (*n* = 11, 25%) and “agree” (n = 11, 25%) (Fig. 5). However, on the posttest, the majority of students selected “agree” (*n* = 28, 65%) (Fig. 5). The paired median response for this question increased from “neither agree nor disagree” (3) to “agree” (4) between the pre- and posttest (p <  0.001) (Table 1).

Additionally, while students ranked competence categories at the lowest level (1) on the pretest and highest level (5) on the posttest, no students ranked competence at the highest level (5) on the pretest or the lowest level (1) on the posttest, further indicating a directional change in competence.

## Discussion

According to the findings in our study, a case-based, interactive online educational module is an effective intervention for improving student knowledge, attitudes, and perceived competence regarding OPM concepts. Prior to our educational intervention, students reflected levels of uncertainty surrounding OP and PM concepts comparable to those observed in similar peer groups [11, 12]. However, posttest results indicated overall student improvement in key knowledge areas such as opioid-sparing PM strategies (alternative medications, first-line treatments for specific pain types), opioid stewardship (PDMP utilization, proper opioid disposal), the opioid epidemic (prescriber contribution, opioid diversion, risk factors for opioid misuse), and appropriate opioid management and prescribing (recommended guidelines, opioid tapering, MME conversions, management of special populations). Improvement in each of these key knowledge areas is imperative for enhancing student preparation as future prescribers since the opioid crisis is a multifactorial epidemic [13] – judicious prescribing extends beyond the quantity of opioid prescribed and encompasses patient-specific considerations such as pain conditions, multimodal analgesia options, opioid-naïve vs. opioid-tolerant dosing, and specific risks for future opioid misuse. Langford et al [14] reported similar results following implementation of an online opioid educational module targeted to improve clinician knowledge and competence in OP for acute pain in hospitalized patients. Similarly designed with interactive content and case-based branching scenarios, clinician reports on follow-up survey indicated reasonable improvement following module intervention in both knowledge and perceived competence on a behavioral PCS constructed to measure OP outcomes comparable to ours [14]. Importantly, we observed similar improvement in all four competence categories from the PCS indicating positive perceived student behavioral changes regarding OP as a result of module intervention.

To further understand the previous exposure to OP experienced by our students, we queried students on sources of opioid training prior to our intervention. We observed a diversity of responses, highlighting the variable exposure to OP and PM experienced by medical students [4]. However, we also observed reported variability within our own medical school curricula, exemplified by the 21% of students that reported no previous opioid training. This intra-institutional disparity suggests that opioid exposure in medical schools may be the result of student elective choices and select patient exposures in addition to variations in curricula between medical schools. This finding strengthens the call for a standardized OPM resource to uniformly prepare students for prescribing responsibilities and reduce the burden on residency programs of addressing gaps in medical education.

While the opioid epidemic is a multifactorial crisis, excessive OP by providers is a key area for harm reduction interventions. The consensus of provider uncertainty surrounding accurate pain assessment and opioid amounts combined with the fear of undermanaging patient pain translates to a significantly increased risk of excessive prescribing [4]. In a survey of primary care physicians conducted by Keller et al. [15], 91.4% of physicians reported prescribing opioids for chronic pain indications, yet 71.5% rated their knowledge and comfort of treatment/management of opioid dependence as low. In turn, this type of prescribing may increase the risk of opioid dependence for the prescription recipient, as well as the risk of leftover opioid pill diversion to an alternative recipient [16, 17]. Unfortunately, after only a single day’s consumption of an opioid prescription, the rate of persistent opioid use is 6% at one year, and escalates to 13.5% if prescription duration reaches a minimum of seven days [18]. The gravity of this dependence risk is well-exemplified in the orthopaedic patient population with 42.3% of surveyed surgeons reporting awareness of development of opioid dependence in at least one patient due to their postoperative prescribing practices [19].

Interventions targeted at the provider-level to reduce excessive prescribing have included initiatives such as state-mandated use of PDMPs for opioid source regulation [20] and the increased publication of studies recommending specialty- and procedure-specific OP guidelines [21–23]. However, expanding the scope of opioid mitigation interventions to improve the preparedness of the student population graduating into the prescriber role is also an imperative [5]. After assessing the experienced curriculum content of opioid-related concepts by medical students interviewing for general surgery during the 2018-2019 application cycle, Di Chiaro et al [12] found that 35.6% of students received no educational instruction on acute PM in their curricula. On further analysis, only 34.4% of these students felt adequately prepared to begin prescribing opioids to surgical patients postoperatively [12]. Importantly, this inadequate preparation of medical students does not change at graduation, but rather traverses their transition into residency where knowledge deficits may have greater consequences. Garcia et al [11] administered a survey to assess opioid knowledge and medical school preparation to first-year internal medicine residents following orientation and found that residents scored an overall 60.7% on opioid knowledge concepts. Furthermore, less than 50% of these residents felt that their medical school curricula had sufficiently prepared them for managing patient pain, and 90% felt inadequately prepared to dose opioids for patients or understand state and federal OP requirements [11].

Although prescription opioid deaths in the U.S. are now due predominantly to fentanyl, and to a lesser extent heroin, prescription opioids remain an important contributor to today’s crisis and the development of OUD for opioid-naïve individuals [24]. While North America overwhelmingly remains the leading consumer of prescription opioids per capita worldwide, increased non-medical use of prescription opioids accompanied by subsequent harms (i.e. overdose and mortality) has begun to emerge in various regions internationally, such as Australia, West and North Africa, and the Middle East [25]. In most European settings, heroin remains the predominant opioid of concern, yet topics such as PM and OUD are underrepresented in medical school curricula worldwide in the context of societal needs [26, 27]. A systematic review performed by Shipton et al. [27] of pain medicine content, teaching, and assessment in medical school curricula internationally revealed an overall lack of curricula focused on PM, with deficits most severe in the United Kingdom and U.S. Internationally, efforts to assess student competency in PM were mostly written examinations lacking clinical application, indicating an important area of improvement for curricula worldwide to address the public health impact of inadequately managed pain [27].

Implementation of our module coincides with a transitional period in medical education worldwide, influenced by the epochal COVID-19 pandemic. While the transition to online learning has been an abrupt and challenging endeavor for medical institutions, this unprecedented event has emphasized the utility of supplementing medical school curricula with tools such as virtual simulations, computer-based models, and asynchronous learning opportunities [28]. The successful adaptation of resources to virtually supplement the learning of large medical student populations [28] creates an important precedent for the virtual dissemination of topics routinely deficient in curricula, such as OP and PM. Given the preliminary success of our module at delivering OPM topics to students, we suggest that this method be further explored as a resource for improved student preparedness surrounding these concepts. The transition to online instruction mandated by the pandemic and subsequent faculty acquisition of improved virtual teaching methods is an indispensable opportunity for unity in combatting the opioid crisis through medical education. The pedagogical methods of human-centered design [7] and case-based learning [8] that we utilized in developing our module should be considered for future modules given their efficacy in creating clinical context that is often deficient in current opioid curricula [4]. We also recommend that this initiative be modified and expanded to other healthcare professional schools that graduate high-volume opioid prescribers such as dental, physician assistant, and nurse practitioner programs [29]. Optimal learning surrounding the complex topic of PM occurs longitudinally throughout the trajectory of healthcare professional training, however a supplemental resource to standardize student preparation is certainly warranted.

### Limitations

To our knowledge, this study is the first to examine the effectiveness of a virtual OPM intervention at the student-level. Accordingly, there are several noteworthy limitations to this study. First, this is a single center study, which potentially limits generalizability. We advocate the need for future studies that will replicate this study design and collect data across multiple institutions. Second, voluntary student participation with a response rate of 19% may not be generalizable to all medical student learners. The voluntary nature of responses (and subsequent lower response rate) has the potential to introduce non-responder bias, indicating that the students voluntarily offering to partake in the study may be substantially different than those who did not respond, and therefore represent a less heterogenous student population. However, our response rate reflects the web-based response rates observed in similarly designed studies [30]. Additionally, student self-reported knowledge, attitudes, and competence may not be valid or reliable enough to measure higher-order outcomes such as patient care or clinical practice. Instead, more objective measures such as direct observation of clinical practice or standardized knowledge assessments should be considered as metrics in future studies to evaluate the effectiveness of this type of intervention in the clinical environment. We emphasize the importance of expanding interventions on this topic to larger student populations and performing longitudinal reviews of prescribing behaviors as students transition into their role as practicing prescribers.

## Conclusion

Implementation of a virtual, interactive module with clinical context is an effective intervention for improving the OPM knowledge, attitudes, and perceived competence of fourth-year medical students. Given the identified deficit of these concepts in medical school curricula and the severity of the U.S. opioid epidemic, this method may be an important resource for augmenting existing medical school curricula and standardizing student exposure to OP across multiple institutions. Creating an accessible and widespread method to effectively prepare our students as future prescribers is crucial for reducing the medical provider contribution to the opioid crisis.

## Supplementary Information

**Additional file 1.**

## Data Availability

The datasets generated and/or analyzed during the current study are available from the corresponding author on reasonable request.

## Abbreviations

U.S.: United States
OP: Opioid prescribing
PM: Pain management
OPM: Opioids and pain management
PDMP: Prescription Drug Monitoring Program
MME: Morphine milliequivalent
MD/MPH: Doctor of Medicine and Master of Public Health
OUD: Opioid use disorder
PCS: Perceived Competence Scale
MAT: Medication-assisted treatment
PCA: Patient-controlled analgesia
COVID-19: Coronavirus disease 2019
