# Supplementary material for: A focus on the future of opioid prescribing: implementation of a virtual opioid and pain management module for medical students
Source: BMC Med Educ. 2022 Jan 6;22:18. doi: 10.1186/s12909-021-03058-z (PMC8733773; doi:10.1186/s12909-021-03058-z)
Supplement: Supplementary file 1 — Additional file 1. [file 12909_2021_3058_MOESM1_ESM.docx]

**Additional File 1**

**Pretest**

*Student Background*

1. How satisfied are you with the amount of opioid prescribing training that you have received in your formal education thus far?
   1. Very unsatisfied
   2. Unsatisfied
   3. Neutral
   4. Satisfied
   5. Very satisfied
2. When did you receive your opioid prescribing training? (select all that apply)
   1. Undergraduate
   2. Medical School
   3. Personal reading
   4. Extracurriculars (research, volunteering, etc.)
   5. Never received any formal training
   6. Other

*General Learning Objectives*

*Please state how strongly you agree or disagree with the following statements:*

1. I understand the risks of opioids in *chronic* pain management
   1. Strongly disagree
   2. Disagree
   3. Neither agree nor disagree
   4. Agree
   5. Strongly agree
2. I understand the benefits of opioids in *chronic* pain management
   1. Strongly disagree
   2. Disagree
   3. Neither agree nor disagree
   4. Agree
   5. Strongly agree
3. I understand when it is appropriate to prescribe opioids for *chronic* pain management
   1. Strongly disagree
   2. Disagree
   3. Neither agree nor disagree
   4. Agree
   5. Strongly agree
4. I understand the risks of opioids in *acute* pain management
   1. Strongly disagree
   2. Disagree
   3. Neither agree nor disagree
   4. Agree
   5. Strongly agree
5. I understand the benefits of opioids in *acute* pain management
   1. Strongly disagree
   2. Disagree
   3. Neither agree nor disagree
   4. Agree
   5. Strongly agree
6. I understand when it is appropriate to prescribe opioids for *acute* pain management
   1. Strongly disagree
   2. Disagree
   3. Neither agree nor disagree
   4. Agree
   5. Strongly agree
7. I am familiar with the types of *opioid* medications used for pain management
   1. Strongly disagree
   2. Disagree
   3. Neither agree nor disagree
   4. Agree
   5. Strongly agree
8. I am familiar with the types of *non-opioid* medications used for pain management
   1. Strongly disagree
   2. Disagree
   3. Neither agree nor disagree
   4. Agree
   5. Strongly agree
9. I understand the role of prescription opioids in the opioid epidemic
   1. Strongly disagree
   2. Disagree
   3. Neither agree nor disagree
   4. Agree
   5. Strongly agree
10. I am familiar with the PDMP and know when to use it
    1. Strongly disagree
    2. Disagree
    3. Neither agree nor disagree
    4. Agree
    5. Strongly agree
11. I am familiar with opioid prescribing guidelines (i.e. dosages and amounts to prescribe)
    1. Strongly disagree
    2. Disagree
    3. Neither agree nor disagree
    4. Agree
    5. Strongly agree

*Case #1*

1. I understand the differences in opioid prescribing for older adults (>65) vs. adults (<65)
   1. Strongly disagree
   2. Disagree
   3. Neither agree nor disagree
   4. Agree
   5. Strongly agree
2. I understand the complexities of pain assessment in patients with dementia
   1. Strongly disagree
   2. Disagree
   3. Neither agree nor disagree
   4. Agree
   5. Strongly agree
3. I understand the differences between nociceptive, neuropathic and inflammatory pain
   1. Strongly disagree
   2. Disagree
   3. Neither agree nor disagree
   4. Agree
   5. Strongly agree
4. I am familiar with the risks and benefits of commonly used pain medications for older adults (>65)
   1. Strongly disagree
   2. Disagree
   3. Neither agree nor disagree
   4. Agree
   5. Strongly agree
5. I am familiar with safe pain management strategies for older adults (>65)
   1. Strongly disagree
   2. Disagree
   3. Neither agree nor disagree
   4. Agree
   5. Strongly agree

*Case #2*

1. I understand the differences in treating nociceptive, neuropathic and inflammatory pain
   1. Strongly disagree
   2. Disagree
   3. Neither agree nor disagree
   4. Agree
   5. Strongly agree
2. I am familiar with first-line treatments and strategies for chronic pain management
   1. Strongly disagree
   2. Disagree
   3. Neither agree nor disagree
   4. Agree
   5. Strongly agree
3. I understand the concept of “opioid diversion”
   1. Strongly disagree
   2. Disagree
   3. Neither agree nor disagree
   4. Agree
   5. Strongly agree
4. I am familiar with safe strategies for disposing of unused opioids
   1. Strongly disagree
   2. Disagree
   3. Neither agree nor disagree
   4. Agree
   5. Strongly agree

*Case #3*

1. I am familiar with the risk factors for patient opioid misuse
   1. Strongly disagree
   2. Disagree
   3. Neither agree nor disagree
   4. Agree
   5. Strongly agree
2. I am familiar with safe management strategies for patients on long-term opioid treatment for chronic pain
   1. Strongly disagree
   2. Disagree
   3. Neither agree nor disagree
   4. Agree
   5. Strongly agree
3. I understand the concept of opioid tapering
   1. Strongly disagree
   2. Disagree
   3. Neither agree nor disagree
   4. Agree
   5. Strongly agree
4. I understand the concept of morphine milligram equivalents (MMEs)
   1. Strongly disagree
   2. Disagree
   3. Neither agree nor disagree
   4. Agree
   5. Strongly agree
5. I understand how to convert morphine milligram equivalents (MMEs)
   1. Strongly disagree
   2. Disagree
   3. Neither agree nor disagree
   4. Agree
   5. Strongly agree
6. I am familiar with the symptoms of an opioid overdose
   1. Strongly disagree
   2. Disagree
   3. Neither agree nor disagree
   4. Agree
   5. Strongly agree
7. I understand the role of naloxone in opioid overdose
   1. Strongly disagree
   2. Disagree
   3. Neither agree nor disagree
   4. Agree
   5. Strongly agree
8. I understand the importance of co-prescribing naloxone with opioids
   1. Strongly disagree
   2. Disagree
   3. Neither agree nor disagree
   4. Agree
   5. Strongly agree

*Case #4*

1. I understand the role of medication-assisted treatment (MAT) in patients with opioid use disorder
   1. Strongly disagree
   2. Disagree
   3. Neither agree nor disagree
   4. Agree
   5. Strongly agree
2. I understand the differences between methadone, buprenorphine, and naltrexone
   1. Strongly disagree
   2. Disagree
   3. Neither agree nor disagree
   4. Agree
   5. Strongly agree
3. I understand the concept of patient-controlled analgesia (PCA)
   1. Strongly disagree
   2. Disagree
   3. Neither agree nor disagree
   4. Agree
   5. Strongly agree
4. I am familiar with the differences in opioid dosing requirements for opioid-tolerant vs. opioid-naïve patients
   1. Strongly disagree
   2. Disagree
   3. Neither agree nor disagree
   4. Agree
   5. Strongly agree

*Case #5*

1. I understand the danger of co-prescribing benzodiazepines and opioids
   1. Strongly disagree
   2. Disagree
   3. Neither agree nor disagree
   4. Agree
   5. Strongly agree
2. I understand the role of multimodal analgesia in pain management
   1. Strongly disagree
   2. Disagree
   3. Neither agree nor disagree
   4. Agree
   5. Strongly agree

*Case #6*

1. I understand the differences in pain medication dosing for the pediatric vs. adult population
   1. Strongly disagree
   2. Disagree
   3. Neither agree nor disagree
   4. Agree
   5. Strongly agree
2. I am familiar with the risks of prescribing opioids to adolescents for pain management
   1. Strongly disagree
   2. Disagree
   3. Neither agree nor disagree
   4. Agree
   5. Strongly agree

*Case #7*

1. I am familiar with pain medications that are safe for pregnant patients
   1. Strongly disagree
   2. Disagree
   3. Neither agree nor disagree
   4. Agree
   5. Strongly agree
2. I am familiar with safe opioid management strategies for pregnant patients
   1. Strongly disagree
   2. Disagree
   3. Neither agree nor disagree
   4. Agree
   5. Strongly agree
3. I understand the postnatal effects of opioids on neonates
   1. Strongly disagree
   2. Disagree
   3. Neither agree nor disagree
   4. Agree
   5. Strongly agree

*Case #8*

1. I am familiar with the concept of pain syndromes in cancer survivors
   1. Strongly disagree
   2. Disagree
   3. Neither agree nor disagree
   4. Agree
   5. Strongly agree
2. I understand the role of opioids in chronic cancer pain
   1. Strongly disagree
   2. Disagree
   3. Neither agree nor disagree
   4. Agree
   5. Strongly agree

*Student Perceived Competence*

1. I am confident in my ability to manage opioids for patient pain
   1. Strongly disagree
   2. Disagree
   3. Neither agree nor disagree
   4. Agree
   5. Strongly agree
2. I am capable of managing opioids for patient pain
   1. Strongly disagree
   2. Disagree
   3. Neither agree nor disagree
   4. Agree
   5. Strongly agree
3. I am able to provide opioid management for patient pain
   1. Strongly disagree
   2. Disagree
   3. Neither agree nor disagree
   4. Agree
   5. Strongly agree
4. I am able to meet the challenge of opioid management for patient pain
   1. Strongly disagree
   2. Disagree
   3. Neither agree nor disagree
   4. Agree
   5. Strongly agree

**Posttest**

*Student Background*

1. How satisfied are you with the amount of opioid prescribing training that you have received in your formal education thus far?
   1. Very unsatisfied
   2. Unsatisfied
   3. Neutral
   4. Satisfied
   5. Very satisfied

*General Learning Objectives*

*Please state how strongly you agree or disagree with the following statements:*

1. I understand the risk of opioids in *chronic* pain management
   1. Strongly disagree
   2. Disagree
   3. Neither agree nor disagree
   4. Agree
   5. Strongly agree
2. I understand the benefits of opioids in *chronic* pain management
   1. Strongly disagree
   2. Disagree
   3. Neither agree nor disagree
   4. Agree
   5. Strongly agree
3. I understand when it is appropriate to prescribe opioids for *chronic* pain management
   1. Strongly disagree
   2. Disagree
   3. Neither agree nor disagree
   4. Agree
   5. Strongly agree
4. I understand the risks of opioids in *acute* pain management
   1. Strongly disagree
   2. Disagree
   3. Neither agree nor disagree
   4. Agree
   5. Strongly agree
5. I understand the benefits of opioids in *acute* pain management
   1. Strongly disagree
   2. Disagree
   3. Neither agree nor disagree
   4. Agree
   5. Strongly agree
6. I understand when it is appropriate to prescribe opioids for *acute* pain management
   1. Strongly disagree
   2. Disagree
   3. Neither agree nor disagree
   4. Agree
   5. Strongly agree
7. I am familiar with the types of *opioid* medications used for pain management
   1. Strongly disagree
   2. Disagree
   3. Neither agree nor disagree
   4. Agree
   5. Strongly agree
8. I am familiar with the types of *non-opioid* medications used for pain management
   1. Strongly disagree
   2. Disagree
   3. Neither agree nor disagree
   4. Agree
   5. Strongly agree
9. I understand the role of prescription opioids in the opioid epidemic
   1. Strongly disagree
   2. Disagree
   3. Neither agree nor disagree
   4. Agree
   5. Strongly agree
10. I am familiar with the PDMP and know when to use it
    1. Strongly disagree
    2. Disagree
    3. Neither agree nor disagree
    4. Agree
    5. Strongly agree
11. I am familiar with opioid prescribing guidelines (i.e. dosages and amounts to prescribe)
    1. Strongly disagree
    2. Disagree
    3. Neither agree nor disagree
    4. Agree
    5. Strongly agree

*Case #1*

1. I understand the differences in opioid prescribing for older adults (>65) vs. adults (<65)
   1. Strongly disagree
   2. Disagree
   3. Neither agree nor disagree
   4. Agree
   5. Strongly agree
2. I understand the complexities of pain assessment in patients with dementia
   1. Strongly disagree
   2. Disagree
   3. Neither agree nor disagree
   4. Agree
   5. Strongly agree
3. I understand the differences between nociceptive, neuropathic and inflammatory pain
   1. Strongly disagree
   2. Disagree
   3. Neither agree nor disagree
   4. Agree
   5. Strongly agree
4. I am familiar with the risks and benefits of commonly used pain medications for older adults (>65)
   1. Strongly disagree
   2. Disagree
   3. Neither agree nor disagree
   4. Agree
   5. Strongly agree
5. I am familiar with safe pain management strategies for older adults (>65)
   1. Strongly disagree
   2. Disagree
   3. Neither agree nor disagree
   4. Agree
   5. Strongly agree

*Case #2*

1. I understand the differences in treating nociceptive, neuropathic and inflammatory pain
   1. Strongly disagree
   2. Disagree
   3. Neither agree nor disagree
   4. Agree
   5. Strongly agree
2. I am familiar with first-line treatments and strategies for chronic pain management
   1. Strongly disagree
   2. Disagree
   3. Neither agree nor disagree
   4. Agree
   5. Strongly agree
3. I understand the concept of “opioid diversion”
   1. Strongly disagree
   2. Disagree
   3. Neither agree nor disagree
   4. Agree
   5. Strongly agree
4. I am familiar with safe strategies for disposing of unused opioids
   1. Strongly disagree
   2. Disagree
   3. Neither agree nor disagree
   4. Agree
   5. Strongly agree

*Case #3*

1. I am familiar with the risk factors for patient opioid misuse
   1. Strongly disagree
   2. Disagree
   3. Neither agree nor disagree
   4. Agree
   5. Strongly agree
2. I am familiar with safe management strategies for patients on long-term opioid treatment for chronic pain
   1. Strongly disagree
   2. Disagree
   3. Neither agree nor disagree
   4. Agree
   5. Strongly agree
3. I understand the concept of opioid tapering
   1. Strongly disagree
   2. Disagree
   3. Neither agree nor disagree
   4. Agree
   5. Strongly agree
4. I understand the concept of morphine milligram equivalents (MMEs)
   1. Strongly disagree
   2. Disagree
   3. Neither agree nor disagree
   4. Agree
   5. Strongly agree
5. I understand how to convert morphine milligram equivalents (MMEs)
   1. Strongly disagree
   2. Disagree
   3. Neither agree nor disagree
   4. Agree
   5. Strongly agree
6. I am familiar with the symptoms of an opioid overdose
   1. Strongly disagree
   2. Disagree
   3. Neither agree nor disagree
   4. Agree
   5. Strongly agree
7. I understand the role of naloxone in opioid overdose
   1. Strongly disagree
   2. Disagree
   3. Neither agree nor disagree
   4. Agree
   5. Strongly agree
8. I understand the importance of co-prescribing naloxone with opioids
   1. Strongly disagree
   2. Disagree
   3. Neither agree nor disagree
   4. Agree
   5. Strongly agree

*Case #4*

1. I understand the role of medication-assisted treatment (MAT) in patients with opioid use disorder
   1. Strongly disagree
   2. Disagree
   3. Neither agree nor disagree
   4. Agree
   5. Strongly agree
2. I understand the differences between methadone, buprenorphine, and naltrexone
   1. Strongly disagree
   2. Disagree
   3. Neither agree nor disagree
   4. Agree
   5. Strongly agree
3. I understand the concept of patient-controlled analgesia (PCA)
   1. Strongly disagree
   2. Disagree
   3. Neither agree nor disagree
   4. Agree
   5. Strongly agree
4. I am familiar with the differences in opioid dosing requirements for opioid-tolerant vs. opioid-naïve patients
   1. Strongly disagree
   2. Disagree
   3. Neither agree nor disagree
   4. Agree
   5. Strongly agree

*Case #5*

1. I understand the danger of co-prescribing benzodiazepines and opioids
   1. Strongly disagree
   2. Disagree
   3. Neither agree nor disagree
   4. Agree
   5. Strongly agree
2. I understand the role of multimodal analgesia in pain management
   1. Strongly disagree
   2. Disagree
   3. Neither agree nor disagree
   4. Agree
   5. Strongly agree

*Case #6*

1. I understand the differences in pain medication dosing for the pediatric vs. adult population
   1. Strongly disagree
   2. Disagree
   3. Neither agree nor disagree
   4. Agree
   5. Strongly agree
2. I am familiar with the risks of prescribing opioids to adolescents for pain management
   1. Strongly disagree
   2. Disagree
   3. Neither agree nor disagree
   4. Agree
   5. Strongly agree

*Case #7*

1. I am familiar with pain medications that are safe for pregnant patients
   1. Strongly disagree
   2. Disagree
   3. Neither agree nor disagree
   4. Agree
   5. Strongly agree
2. I am familiar with safe opioid management strategies for pregnant patients
   1. Strongly disagree
   2. Disagree
   3. Neither agree nor disagree
   4. Agree
   5. Strongly agree
3. I understand the postnatal effects of opioids on neonates
   1. Strongly disagree
   2. Disagree
   3. Neither agree nor disagree
   4. Agree
   5. Strongly agree

*Case #8*

1. I am familiar with the concept of pain syndromes in cancer survivors
   1. Strongly disagree
   2. Disagree
   3. Neither agree nor disagree
   4. Agree
   5. Strongly agree
2. I understand the role of opioids in chronic cancer pain
   1. Strongly disagree
   2. Disagree
   3. Neither agree nor disagree
   4. Agree
   5. Strongly agree

*Student Perceived Competence*

1. I am confident in my ability to manage opioids for patient pain
   1. Strongly disagree
   2. Disagree
   3. Neither agree nor disagree
   4. Agree
   5. Strongly agree
2. I am capable of managing opioids for patient pain
   1. Strongly disagree
   2. Disagree
   3. Neither agree nor disagree
   4. Agree
   5. Strongly agree
3. I am able to provide opioid management for patient pain
   1. Strongly disagree
   2. Disagree
   3. Neither agree nor disagree
   4. Agree
   5. Strongly agree
4. I am able to meet the challenge of opioid management for patient pain
   1. Strongly disagree
   2. Disagree
   3. Neither agree nor disagree
   4. Agree
   5. Strongly agree

*Feedback*

1. Please provide any additional feedback that you think would be helpful for improving this course for future students.
